# Antifungal and antibacterial effects of newly created lactic acid bacteria associations depending on cultivation media and duration of cultivation

**DOI:** 10.1186/s12866-019-1475-x

**Published:** 2019-05-17

**Authors:** Lusine Matevosyan, Inga Bazukyan, Armen Trchounian

**Affiliations:** Department of Biochemistry, Microbiology and Biotechnology, Faculty of Biology, Yerevan State University, 1 Alex Manoogian Str, 0025 Yerevan, Armenia

**Keywords:** Lactic acid bacteria, Antifungal activity, Antibacterial activity, LAB associations

## Abstract

**Background:**

The newly created associations of lactic acid bacteria (LAB) isolated from Armenian dairy products (yoghurt, sour cream and different varieties of cheese), as well as from the gastrointestinal tract of honeybees were screened according to their antifungal and antibacterial activity.

**Results:**

LAB strains were mixed at equal proportions (1:1) according to mathematical planning of experiments. Antifungal and antibacterial effects of different combinations (associations) were determined in different media, employing well-diffusion and total diffusion into agar methods. A number of fungal and bacterial test-organisms, including pathogenic ones, were used. Pure LAB strain cultures were used as a control. The antifungal effect of the most active strain *Lactobacillus rhamnosus* MDC 9661 in the associations with other LAB strains was partly decreased. At the same time, some mixed LAB cultures in DeMan, Rogosa and Sharpe (MRS) medium demonstrated significant antibacterial activity against wide spectra of test-organisms only in the case of simultaneous cultivation of LAB strains. On the other hand, in the case of different LAB strains cultivated in MRS with 24-h time break between mix formations by different strains, no inhibitory activity was revealed. But the inhibitory effect of many LAB associations against test-organisms was significantly increased in the case of separated cultivation in milk.

**Conclusion:**

The inhibitory effect of mixed LAB associations showed stronger dependence on the cultivation media and on the duration of cultivation with respect to each other. The co-cultivation of some strains, like *L. rhamnosus* MDC 9661, could lead to changed antagonistic activity. Consequently, the results are significant for creation and further investigation of LAB associations, as effective probiotics, and for their probable application in the production of antimicrobial preparations.

## Background

Various pathogenic microorganisms, including fungi, which contaminate the nutrients of humans and animals, can be potentially dangerous agents and cause the loss of money [1]. The reducing contamination by various pathogenic bacteria, molds and yeasts in the production and storage of food and feed is of paramount importance, and the most interesting task is to develop the efficient and safe possibilities for this purpose. The use of substrates of both natural origin and well- investigated manufacturers are a key rule for choosing new agents. Lactic acid bacteria (LAB) best meet these requirements. In recent years, special interest in the antagonistic activity of bacterial communities is increased.

LAB have been used in the domestic food production for centuries not only because of they were tasty, but also because they allowed food to be stored for a long period of time. A lot of representatives of this group showed high antagonistic activity. The group of LAB is composed of the representatives of such genera, as *Lactobacillus, Lactococcus, Streptococcus, Leuconostoc*, *Pediococcus, Enterococcus, Oenococcus, Weissella* and others [2]. Of great interest is the genus *Lactobacillus* because of multiple antagonistic activities of its representatives. The list of antimicrobial metabolites synthesized by LAB includes organic acids, hydrogen peroxide, various lipids and bacteriocins [3]. LAB as the best probiotics were granted the status of Generally Regarded As Safe (GRAS) by the Food and Drug Administration (FDA) [4], and their benefits for human and animal gastrointestinal tract and immune system have been well known. However, the lack of the toxic effects of their metabolites is still under investigation [5, 6].

The crucially different from each other ecological-geographic conditions of Armenia lead to the development of unique communities of LAB in national food products. Several data about the antimicrobial effects of mixed LAB cultures against food-borne pathogens have been published [7–11]. However, their dependence on different factors, including media composition and duration of cultivation, was not shown clearly. It is very important to create correct associations, in which all strains will show the synergistic effect not only in simultaneous growth but also during the synthesis of active components. Various scenarios are possible in these associations: competition for nutrients, inhibition of one strain by other antibacterial components, inhibition of necessary actions due to the use of bacterial components instead of preliminary nutrients, etc. Unfortunately, less data on the pathway of metabolism in microbial associations is known. So this is the first problem to be solved. The study of mixed LAB cultures would explain the various microbial-microbial interactions that can be used in the production of food and drugs.

The aim of this work was the creation of various LAB associations from the previously studied strains which have strong antimicrobial effects separately. The dependence of antifungal and antibacterial effects of the LAB combinations on the composition of nutrient media and cultivation has been studied.

## Results

### Antifungal activity of LAB associations

The results of the antifungal effects of individual LAB strains were presented in previous studies [12], and were also increased in Table 1. A study of the antifungal activity of 15 different LAB associations showed that they have almost the same inhibitory effects on various types of mold and yeast (Table 2). Interestingly, inhibitory effect of *L. rhamnosus* MDC 9661 in combination with other LAB strains (VKPM B-3809, RIN-2003-Ls, INRA-2010-4.2, INRA-2010-5.2 and INR-2010-Tsov-G-St) was partly decreased (see Table 2, Mixes 1–5). Particularly, *L. rhamnosus* MDC 9661 has partly lost its activity against *P. aurantioviolaceum* and *G. candidum* in the mixture with *S. thermophilus* VKPM B-3809, as well as against *P. aurantioviolaceum* and *A. flavus* in the mixture with RIN-2003-Ls*.* The associations of MDC 9661 with INRA-2010-4.2 and INRA-2010-5.2 couldn’t inhibit the growth of *P. aurantioviolaceum, T. viride* and *A. flavus.* Finally, the mixture of MDC 9661 with INR-2010-Tsov-G-St did not show any activity against *A. flavus*. None of the associations can inhibit the growth of yeast. And only the combination of *L. rhamnosus* MDC 9661 with *S. thermophilus* VKPM B-3809 could inhibit the growth of *A. flavus*. Thus, we can conclude that the creation of associations with different strains of LAB and *L. rhamnosus* MDC 9661 is impractical; because in all associations, inhibition of the antifungal activity of MDC 9661 was observed.

**Table 1:** Antifungal activity of LAB isolates

Fungi LAB strain	*M. plumbeus*	*P. aurantio-violaceum*	*Penicillum*spp.	*F. oxysporum*	*C. herbarum*	*T. viride*	*G. candidum*	*A. flavus*
MDC 9661	**+**	**+**	**+**	**+**	**+**	**+**	**+**	**+/−**
RIN-2003-Ls	**–**	**–**	**–**	**+**	**+**	**–**	**–**	**–**
MDC 9632	**–**	**–**	**–**	**+**	**+**	**–**	**–**	**–**
MDC 9633	**–**	**–**	**–**	**+/−**	**–**	**–**	**–**	**–**
VKPM B-3809	**–**	**–**	**–**	**+**	**–**	**–**	**–**	**–**
INR-2010-Tsov-G-St	**–**	**–**	**–**	**+**	**–**	**–**	**–**	**–**

(+) - presence of antifungal activity which means the inhibition of 10^4^ fungi spores, (−) - absence of antifungal activity, (+/−) - elongation of spore generation

**Table 2:** Antifungal activity of LAB associations

Fungi Mix (1:1)	*M. plumbeus*	*P. aurantio-violaceum*	*Penicillum*spp.	*F. oxysporum*	*C. herbarum*	*T. viride*	*G. candidum*	*A. flavus*
1	**+**	**–**	**+**	**+**	**+**	**+**	**–**	**+**
2	**+**	**–**	**+**	**+**	**+**	**+**	**+**	**–**
3	**+**	**–**	**+/−**	**+**	**+**	**–**	**+**	**–**
4	**–**	**–**	**+**	**+**	**+**	**–**	**+**	**–**
5	**+**	**+**	**+**	**+**	**+**	**+**	**+**	**–**
6	**–**	**–**	**–**	**+**	**+**	**–**	**–**	**–**
7	**–**	**–**	**–**	**–**	**–**	**–**	**–**	**–**
8	**–**	**–**	**–**	**–**	**–**	**–**	**–**	**–**
9	**–**	**–**	**–**	**+**	**+/−**	**–**	**–**	**–**
10	**–**	**–**	**–**	**+**	**–**	**–**	**–**	**–**
11	**–**	**–**	**–**	**–**	**+/−**	**–**	**–**	**–**
12	**–**	**–**	**–**	**+**	**–**	**–**	**–**	**–**
13	**–**	**–**	**–**	**–**	**–**	**–**	**–**	**–**
14	**–**	**–**	**–**	**–**	**+/−**	**–**	**–**	**–**
15	**–**	**–**	**–**	**+/−**	**–**	**–**	**–**	**–**

(+) - presence of antifungal activity which means the inhibition of 10^4^ fungi spores, (−) - absence of antifungal activity, (+/−) - elongation of spore generation

### Antibacterial activity of LAB associations

**Fig. 1:**
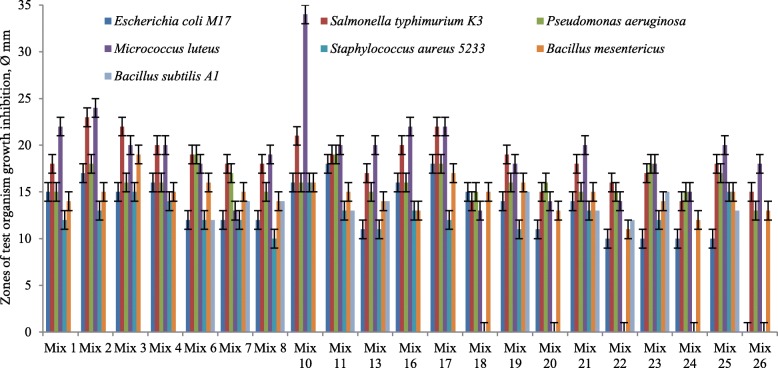
Antibacterial activity of LAB communities in modified MRS-broth. Simultaneous cultivation (strains included in the same mix have the same cultivation duration)

The results of the antibacterial activity of individual LAB strains are presented in Table 3. Mixed cultures showed a significant difference depending on the type of cultivation and the type of growth medium. Interestingly, some LAB associations showed significant antibacterial activity in MRS against *E. coli* VKPM-M17, *B. mesentericus* WT, potentially pathogenic *S. typhimurium* MDC 1759, *P. aeruginosa* WT272786 and *S. aureus* MDC 5233 in the case of simultaneous cultivation of LAB strains (Fig. 1).

**Table 3:** Antibacterial activity of LAB isolates

Test-organism LAB strain	Medium	*E. coli* M17	*Salmonella typhimurium 1759*	*M. luteus*	*P. aeruginosa*	*S. aureus*	*B. mesentericus*	*B. subtilis*
MDC 9661	Skim milk	13^*^	16	18	13	17	–	–
	MRS	11	14	10	10	18	10	–
RIN-2003-Ls	Skim milk	12	15	18	10	18	–	11
	MRS	12	12	9	9	–	11	–
MDC 9632	Skim milk	12	13	17	–	15	11	11
	MRS	12	12	9	11	–	11	–
MDC 9633	Skim milk	12	15	18	10	16	10	–
	MRS	11	13	12	10	11	10	–
VKPM B-3809	Skim milk	10	12	16	–	12	–	–
	MRS	12	12	9	10	18	10	–
E.d	Skim milk	10	12	17	–	12	–	–
	MRS	12	11	9	10	20	10	–
B7	Skim milk	–	12	17	9	15	9	–
	MRS	11	15	9	11	13	16	–

^*^Zones of test organism growth inhibition, Ø mm

**Fig. 2:**
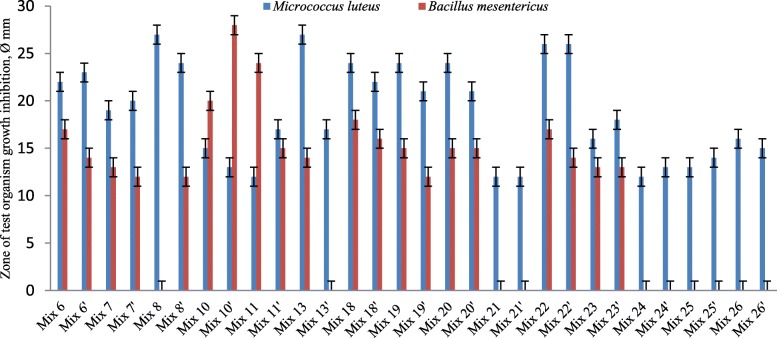
Antibacterial activity of LAB communities in modified MRS-broth. Cultivation separated by time (strains included in the same mix have different cultivation duration)

No inhibitory activity was observed against potential pathogenic strains in the case of time-spaced LAB cultivation in MRS. In this case, antibacterial effect was determined only against *M. luteus* WT and *B. mesentericus* WT (Fig. 2). Thus, the simultaneous cultivation of LAB strains can stimulate the production of antibacterial substances, enlarging their antibacterial activity.

**Fig. 3:**
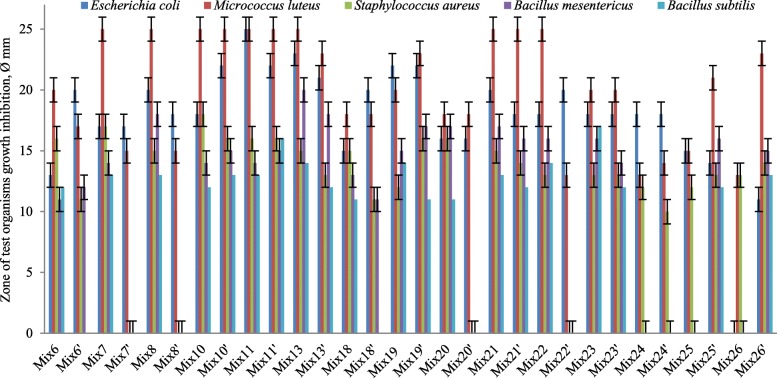
Antibacterial activity of LAB communities in milk. Cultivation separated by time (strains included in the same mix have different cultivation duration)

Compared with cultivation in MRS, the inhibitory effect of many LAB associations was significantly increased in milk against *E. coli* VKPM-M17, *S. aureus* MDC 5233*, B. mesentericus* WT in the case of time-spaced strain cultivation (Fig. 3). The same composition of media and cultivation type affected the antibacterial activity of mixed LAB cultures. Interestingly, some mixed cultures in milk showed an inhibitory effect even against *B. subtilis* WT-A1 strain, but no activity was detected against potential pathogenic test-organisms. The simultaneous cultivation of LAB was not investigated in milk, because the strain MDC 9661 could not grow in milk within 24 h.

## Discussion

The study of antimicrobial activity of LAB strains, especially their mixed cultures, becomes more and more promising. Unfortunately, there are not so many published works about antimicrobial effects of mixed LAB cultures; only a few data were published by different groups of researchers. Particularly, LAB associations suppressed the growth of pathogens during preparation of *borde*. Borde is a traditional Ethiopian fermented low-alcohol beverage [7]. During the *borde* fermentation a lot of outside microorganisms can contaminate the product. The authors showed the presence of antibacterial activity of *borde* LAB strains against all these bacteria. It should be noted that the amount of test microorganisms was increased by 3 log during 8 h cultivation in in supernatants that did not contain LAB cells. Interestingly, during simultaneous LAB cultivation and test organisms, the colony forming units (CFU) of LAB was increased by the same amount under these conditions.

Other authors investigated the antibacterial activity of LAB mixtures which had been composed of six different strains. All these strains were isolated from the Ethiopian fermented milk *ergo* [10]. They showed that the growth of *P. aeruginosa*, *S. aureus* and *Sh. boyidii* was completely inhibited during 5, 6, and 7 days, respectively, after addition of the newly created LAB mixture into *ayib*.

In another work LAB strains were cultivated separately, but their cell-free cultural liquid was used for investigation of antibacterial activity [11]. The highest antimicrobial activity of cell suspension was determined in MRS after 18 h incubation. The results indicated that *S. thermophilus* with *L. bulgaricus* had the highest antibacterial effect against *S. aureus* with a zone inhibition of 10.5 ± 0.35 mm and for *E. coli* with 4 ± 0 mm. On the other hand, our results showed the overproduction of inhibitory agents of LAB associations in the case of simultaneous cultivation in MRS. Simultaneously the antibacterial activity of various mixtures consisted of *L. rhamnosus* MDC 9661 and other LAB strains was increased from 10 ± 2 mm of growth inhibition zone up to 16 ± 1 mm in average against *P. aeruginosa* WT272786. Otherwise the overproduction of antibacterial components observed at the time spaced system during cultivation in milk. This may be due to production of some components, which are synthesized during the primary metabolism and used by the other bacteria, as a stimulator of antibacterial activity. This process occurs only in the presence of dairy components.

Sometimes LAB synergism can reduce their antimicrobial effect. Specifically, Domínguez-Manzano and Jiménez-Díaz [8] showed that bacteriocin production is significantly reduced in LAB co-cultures. Bacteriocins can be a good source of amino acids, and some LAB strains use these antibacterial components, as nutrients. This is possible due to the high proteolytic activity of strains. This mechanism allows them to survive at the presence of bacteriocin and can lead to inhibition of antagonistic activity [8]. At the same time, the activation of growth of both symbiotic strains can be observed. Similarly, the same results were obtained during our experiments, when the antifungal activity of MDC 9661 combinations with other LAB was partially decreased.

Interesting results were obtained by other researchers in the case of antifungal activity of LAB associations as well as associations of LAB and lactose fermenting yeasts [9]. Specifically, they showed that these associations have antagonistic activity against *Penicillium notatum, Penicillium* spp. and *Cephalosporium humicola* [9].

For centuries, Armenians have been preparing national food which has a stimulating effect on human and animal immune system, health, etc. [13]. Traditional Armenian food is rich in proteins and essential nutrients. For the production of such kind of food the rural population separated and cultivated unique LAB associations for centuries. The selection was made on the basis of their antimicrobial, proteolytic and antifungal activities. The antibacterial and proteolytic activity of some isolated strains had been shown before [14]. The antifungal effects of many LAB strains were investigated against different kinds of moulds in the previous study [12].

The antimicrobial components synthesized by LAB are in demand in industry as safe and specific bio-preservatives [15]. Many bacteriocins produced by this group of bacteria have successfully proved to be preservatives for meat products, sea food, dairy and cereal products, fermented vegetables and fruits [16]. The main approaches to use of bacteriocins, as well as intact LAB strains with high inhibitory activity are extending the shelf life of foods, immobilizing them in packing materials and adding them to other preservatives. For the confirmation of the bacteriocineous nature of investigated LAB antibacterial components, the multiple following experiments should be done. Because we used previously investigated LAB strains in all mixtures, we can only assume that in the combinations they synthesized some antibacterial components with proteinaceous nature [14, 17].

## Conclusions

Thus, it can be concluded that LAB associations revealed a stronger inhibitory effect in milk in the case of time-spaced cultivation. Co-cultivation of some strains, such as *L. rhamnosus* MDC 9661, could lead to the inhibition of its antagonistic activity. Of course, for the confirmation of this and understanding of the mechanisms of counteraction between LAB strains in associations, continual and more detailed research is required.

## Methods

### Objects of investigation

The different LAB strains isolated from traditional Armenian dairy product matsoun, cheeses and honeybees’ gastrointestinal tract were used as the objects of this investigation: *Lactobacillus rhamnosus* R-2002 (the accession number is KY054594 and submitted in GenBank) deposited at Microbial Depository Center (MDC) (WDM803) (‘Armbiotechnology’ Scientific and Production Center, National Academy of Sciences of Armenia, Yerevan, Armenia) under the number MDC 9661, *Lactobacillus delbrueckii* subsp. *bulgaricus* (RIN-2003-Ls)*, L. delbrueckii* subsp. *lactis* INRA-2010-4.2 and *L. delbrueckii* subsp. *bulgaricus* INRA-2010-5.2 under the code numbers MDC 9632 and MDC 9633, respectively [18]*, Enterococcus faecium* INR-2010-Tsov-G-St, *Streptococcus thermophilus* VKPM B-3809*, Enterococcus durans* (E.d - provided by Institut Nationale de la Recherche Agronomique, Nantes, France, INRA), *Lactobacillus* spp. (B7 - isolated from honeybees).

### Creation of LAB associations

All associations were created in accordance with the method explained by Matevosyan et al. [20]. The LAB strains were cultivated in modified MRS broth (10 g l^− 1^ meat extract, 10 g L^− 1^ poly-peptone, 5 g L^− 1^ yeast extract, 20 g L^− 1^ glucose, 2 g L^− 1^ ammonium citrate, 0.2 g L^− 1^ MgSO_4_, 0.05 g L^− 1^ MnSO_4_, 1 g L^− 1^ Tween 80, 0.8% agar, sterilization at 1 atm., 15 min) at 37 °C during 24 h. For the same purpose, the 10% milk was used (the milk powder was produced by “Katnarat” LLC, Armenia) [21].

### Determination of antifungal activity

To determine the antifungal activity, the six most active LAB strains were selected, and from these strains 15 different combinations were created according to Bazukyan et al. [19] and Matevosyan et al. [20]. The antifungal activity of *E. durans* was not studied, since it is the reference strain from INRA (Nantes, France), the antifungal activity of which was shown by Ahmadova et al. [22]. Strain B7 was not included in creation of associations because it was not active. The antifungal properties of LAB associations were determined by both well-diffusion method and total diffusion into agar. It should be mentioned, that all experiments were carried out using mod MRS media since it was suitable for both the growth of fungi (molds) and the growth of LAB. Various species of molds and yeasts were used as test-organisms: *Mucor plumbeus*, *Geotrichum candidum, Fusarium oxysporum*, *Cladosporium herbarum* (isolated from spoiled food and provided by Biopolymers interaction assemble, function and interaction of proteins laboratory (FIPL), INRA) [22], *Aspergillus flavus, Penicillium aurantioviolaceum, Penicillium* spp. and *Trichoderma viride* (isolated from spoiled food and provided by Dr. K. Grigoryan, Yerevan State University, Yerevan, Armenia), *Candida albicans* 301 (isolated from clinical material), *Debaryomyces hansenii.* The well-diffusion method was carried out according to the method provided in [23] with using as the cultivation media Sabouraud with 0.9% w v^− 1^ of agar. 50 μl of each LAB cultural liquid (LAB had been grown in modified MRS broth at 37 °C during 24 h) was added to wells (2 different LAB strains were mixed at equal proportions). The presence of antifungal activity was determined by the absence of fungal/yeasts growth around of well (qualitative test). Using diffusion into agar, 250 μL of each overnight LAB (2 different LAB strains were mixed at equal proportions) cultural liquid was added to small (7 mL) Petri dishes and covered by modified MRS agar. The suspension of fungal/molds spores (the amount was 10^4^ per ml) was dropwise applied on the surface of media after 48 h LAB cultivation. The suspensions of fungal spores were prepared according to Bazukyan et al. [19]. The antifungal activity was detected in the absence of fungal/molds growth on the surface of the medium (quantitative test). The antibacterial and antifungal activities of LAB mixtures were compared with the results of the initial strains.

### Determination of antibacterial activity

Seven LAB strains were selected according to high antibacterial activity, which was studied previously [14, 18]. The strain *E. faecium* INR-2010-Tsov-G-St did not show any antibacterial activity, that is why it was not included in the combinations.

The antibacterial activity of 21 different LAB associations was determined. Antibacterial properties of LAB mixed cultures were studied both in modified MRS-broth and milk by two methods of cultivation (simultaneous cultivation of LAB strains and cultivation of these strains separated by time at 37 °C) by agar well diffusion method [20, 23]. Different Gram-positive and Gram-negative representatives of various genera were used for detection of antibacterial activity: *Staphylococcus aureus* MDC 5233 (MDC, Armenia), *Bacillus mesentericus* WT, *B. subtilis* WT-A1 (isolated from a soil sample) and *Micrococcus luteus* WT (isolated from an air sample), *Escherichia coli* VKPM-M17 (Russian National Collection of Industrial Microorganisms, Institute of Genetics and Selection of Industrial Microorganisms, Moscow, Russia), *Salmonella typhimurium* MDC 1759 and *Pseudomonas aeruginosa* WT272786 (isolated from clinical material and provided by “Prom-Test” LLC, Yerevan, Armenia). 100 μL of the mixtures was added to well. Diffusion of antibacterial substances happened during 30 min at room temperature. The diameters of test-organisms growth inhibition zones were measured after 24 h. As a positive result, at least 2 mm in diameter, a net zone of inhibition was detected, as a positive result.

Simultaneous cultivation was carried out by mixing of each 2 separate LAB strains (0.5%) in the same growth medium (modified MRS-broth) and cultivating together at 37 °C during 24 h. The CFU of each initial LAB strain was adjusted to 10^8^ before mixing. The agar well-diffusion method, which described above, has also been used in these experiments. The second way was time-spaced LAB strains cultivation (strains have different cultivation duration). At the first time, the first LAB strain was cultivated in mod MRS (or milk) during 24 h at 37 °C and then mixed with the second strain and cultivated again under the same conditions. So, the first LAB strain was cultivated during 48 h, and the second strain was cultivated during 24 h. Then the places of strains were changed. In each case the antibacterial activity of associations was compared with the same activity of LAB pure cultures.

### Data processing

All data were averages of three independent experiments. The standard errors were determined using Software Excel 2013.

## Abbreviations

BIA FIPL: Biopolymers Interaction Assemble, Function and Interaction of Proteins Laboratory INRA National Research Institute of Agronomy
LAB: lactic acid bacteria
MDC: Microbial Depository Center
MRS: DeMan, Rogosa and Sharpe
VKPM: Russian National Collection of Industrial Microorganisms, Institute of Genetics and Selection of Industrial Microorganisms
